# Supplementary material for: Early-life diet does not affect preference for fish in herring gulls (Larus argentatus)
Source: PeerJ. 2024 Jul 11;12:e17565. doi: 10.7717/peerj.17565 (PMC11246621; doi:10.7717/peerj.17565)
Supplement: Supplemental Information 1 — Colours represent the two treatment groups (Marine group n = 14 chicks, Terrestrial n = 13). The solid lines represent the medians (smoothed for visual only, lambda = 20) for both treatment groups and the dashed lines present smoothed IQRs (0.25 and 0.75, lambda = 20). [file peerj-12-17565-s001.pdf]

<u>Supplementary Materials Figure S1: Early-life diet does not affect preference for fish in herring gulls (*Larus argentatus*)</u>

Emma Inzani[1*], Dr Laura Kelley[1], Dr Robert Thomas[2], Dr Neeltje Boogert[1]

[1]Centre for Ecology and Conservation, University of Exeter, Penryn, Cornwall, UK
[2] Organisms and Environment Division, Cardiff School of Biosciences, Cardiff University, Cardiff, Wales, UK

***Corresponding author:** eli204@exeter.ac.uk

<u>Chick Weight</u>

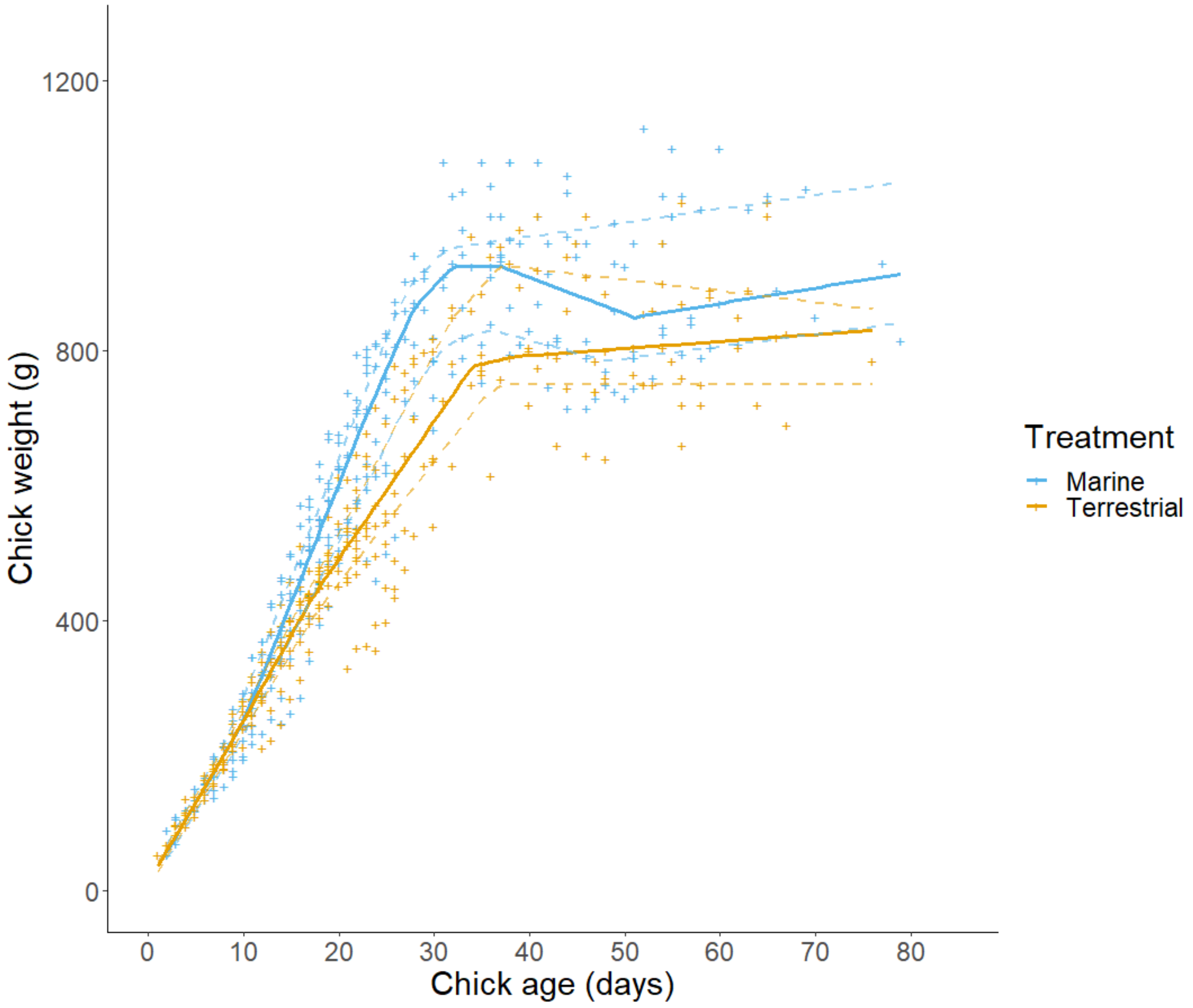

Supplementary Figure S1: Individual chick weights by chick age. Colours represent the two treatment groups (Marine group n = 14 chicks, Terrestrial n = 13). The solid lines represent the medians (smoothed for visual only, lambda = 20) for both treatment groups and the dashed lines present smoothed IQRs (0.25 and 0.75, lambda = 20).

<u>Chick growth graphs</u>

Our study chicks' growth appears to follow previous studies observations (Kadlec, Drury, and Onion 1969; Spaans 1971), with chicks in weight and tarsus measurement (as a proxy for skeletal size) appear to show sigmoidal growth curves (Supplementary materials Figures S1 and S2 for chick

weight and tarsus respectively) that show exponential growth between the approximate ages of 8 – 30 days old before growth slows to a plateau.

Kadlec, John A, William H Drury, and Daniel K Onion. 1969. 'Growth and Mortality of Herring Gull Chicks'. *Journal of Field Ornithology* 40 (3): 222–33.

Spaans, A. L. 1971. 'On the Feeding Ecology of the Herring Gull Larus Argentatus Pont. in the Northern Part of the Netherlands'. *Ardea* 38–90: 73–188. https://doi.org/10.5253/arde.v59.p73.
