# Supplementary material for: Early-life diet does not affect preference for fish in herring gulls (Larus argentatus)
Source: PeerJ. 2024 Jul 11;12:e17565. doi: 10.7717/peerj.17565 (PMC11246621; doi:10.7717/peerj.17565)
Supplement: Supplemental Information 3 [file peerj-12-17565-s003.docx]

**Supplementary Materials Table S1: Early-life diet does not affect preference for fish in herring gulls (*Larus argentatus*)**

**Emma Inzani^1*^, Dr Laura Kelley^1^, Dr Robert Thomas^2^, Dr Neeltje Boogert^1^**

^1^Centre for Ecology and Conservation, University of Exeter, Penryn, Cornwall, UK

^2^ Organisms and Environment Division, Cardiff School of Biosciences, Cardiff University, Cardiff, Wales, UK

***Corresponding author:** eli204@exeter.ac.uk

| Table S1: Calorific and nutritional values from nutritional analyses | | | | | |
| --- | --- | --- | --- | --- | --- |
|  | Calorific content kJ/100g | Protein g/100g | Fat g/100g | Carbohydrates g/100g | Moisture g/100g |
| Brown Bread^1^ | 986 | 12 | 2.9 | 37 | NA |
| Cat Food ^1^  ^2^ Controlling for moisture | 95.2 calculated from protein  540 calculated from protein | 6.5  37 | 4  22 | 3.5  No Information | 82.5  0 |
| Cooked Mussels^1^ | 457 | 15.6 | 2.3 | 5.9 | NA |
| Sprats^3^ | 718 | 18 | 11 | 0 | 66.3 |
| Mackerel^3^ | 968 | 18 | 17.9 | 0 | 61.9 |
| ^1^ Nutritional values as stated on the packaging.  ^2^ Cat food relative nutritional values without 82.5% moisture constituent (jelly that wasn’t consumed) per 100g. calculation Sources: (Davies et al. 2017),  <https://www.petmd.com/cat/nutrition/do-cats-need-high-protein-cat-food>  ^3^ Source for average nutritional values: McCance and Widdowson’s composition of foods integrated dataset (raw mackerel filleted, raw sprats whole):  <https://www.gov.uk/government/publications/composition-of-foods-integrated-dataset-cofid> | | | | | |

Davies, M., R. Alborough, L. Jones, C. Davis, C. Williams, and D. S. Gardner. 2017. ‘Mineral Analysis of Complete Dog and Cat Foods in the UK and Compliance with European Guidelines’. *Scientific Reports* 7 (1): 17107. https://doi.org/10.1038/s41598-017-17159-7.
