# Supplementary material for: Early-life diet does not affect preference for fish in herring gulls (Larus argentatus)
Source: PeerJ. 2024 Jul 11;12:e17565. doi: 10.7717/peerj.17565 (PMC11246621; doi:10.7717/peerj.17565)
Supplement: Supplemental Information 4 [file peerj-12-17565-s004.docx]

**Supplementary Materials Table S2: Early-life diet does not affect preference for fish in herring gulls (*Larus argentatus*)**

**Emma Inzani^1*^, Dr Laura Kelley^1^, Dr Robert Thomas^2^, Dr Neeltje Boogert^1^**

^1^Centre for Ecology and Conservation, University of Exeter, Penryn, Cornwall, UK

^2^ Organisms and Environment Division, Cardiff School of Biosciences, Cardiff University, Cardiff, Wales, UK

***Corresponding author:** eli204@exeter.ac.uk

| Table S2: Results of two-way Intraclass Correlation Coefficients (ICCs) testing the repeatability of behavioural measures within and between individual chicks. | | | | | | |
| --- | --- | --- | --- | --- | --- | --- |
|  | **Agreement or Consistency** | **No. Trials (raters)** | **No. Subjects** | **ICC** | **95% CL** | **P value** |
| First food preference | Agreement | 4 | 10 | -0.13 | -0.15 - 0.061 | 0.96 |
| First food preference – excluding 35 day test | Agreement | 3 | 21 | -0.031 | -0.13 - 0.16 | 0.65 |
| Second food preference | Agreement | 4 | 5 | 0.076 | -0.13 - 0.62 | 0.21 |
| Second food preference – excluding 35 day test | Agreement | 3 | 17 | -0.078 | -0.213 - 0.39 | 0.25 |
| First food preference | Consistency | 4 | 10 | -0.28 | -0.314 - -0.171 | 0.99 |
| First food preference – excluding 35 day test | Consistency | 3 | 21 | -0.056 | -0.25 - 0.24 | 0.65 |
| Second food preference | Consistency | 4 | 5 | 0.15 | -0.17 - 0.77 | 0.22 |
| Second food preference – excluding 35 day test | Consistency | 3 | 17 | 0.092 | -0.16 - 0.44 | 0.25 |
